# Treatment Effects and Treatment Time in Adolescents With Crowded and Displaced Teeth Treated With Fixed Appliance Systems Without Extractions: A Multi‐Centre Randomised Controlled Trial

**DOI:** 10.1111/ocr.70005

**Published:** 2025-07-23

**Authors:** Linda Bokander Matilainen, Kristina Johansson, Michal Wiaderny, Anna Brechter, Sofia Petrén, Vincenzo D'Antò, Liselotte Paulsson

**Affiliations:** ^1^ Faculty of Odontology, Malmö University Malmö Sweden; ^2^ Department of Orthodontics Östersund Hospital Östersund Sweden; ^3^ Department of Orthodontics Folktandvården Dalarna Falun Sweden; ^4^ Private Orthodontic Practice, Bernhold Ortodonti Helsingborg Sweden; ^5^ Department of Neurosciences, Reproductive Sciences and Oral Sciences, School of Orthodontics University of Naples Federico II Naples Italy

**Keywords:** adolescents, malocclusion, orthodontic appliances, RCT, treatment outcome

## Abstract

**Objectives:**

To assess and compare treatment effects and treatment time in adolescents with crowded and displaced teeth during and after non‐extraction treatments using a passive self‐ligating bracket system (PSLB) and a conventional bracket system (CB).

**Materials and Methods:**

Consecutive adolescents (12–17 years old) with crowded and displaced teeth were randomised through computer generated block randomisation in a two‐arm parallel‐group design, to non‐extraction fixed appliance treatment with either PSLB (Damon Q, *n* = 62) or CB (Victory, *n* = 70) at four orthodontic clinics. Study models and cephalograms were collected at baseline, post‐alignment and post‐treatment. Primary outcome was assessed with weighted Peer Assessment Rating index (wPAR) and secondary outcomes with Little's Irregularity Index (LII), and as transversal width (mm), incisor inclination (°), position (mm) and treatment time (months).

**Results:**

Intergroup differences in wPAR with mean score reductions of −24.07 (CB) and −22.11 (PSLB), LII upper/lower arch reductions of −9.02/−5.96 (CB) and −8.44/−5.29 (PSLB), and mean treatment times of 21.74 (CB) and 24.35 (PSLB) months were non‐significant (NS). Lateral expansion was greater in the PSLB group during alignment; mean intergroup maxillary differences (MD) were 1.35 at inter‐canines, 1.21 mm at first inter‐premolars, and mandibular MD were 0.75 at first, 0.76 mm at second inter‐premolars (*p* ≤ 0.019). Greater incisor proclination was observed in the CB group (L1NB, MD = 2.28°; ILiML, MD = 2.23°) (*p* ≤ 0.016). Intergroup differences post‐treatment were NS.

**Conclusion:**

Great occlusal treatment outcomes were observed in both groups with no significant difference in treatment time. Although the expansion patterns differed during alignment, no significant intergroup difference remained after treatment.

**Trial Registration:**

The protocol is registered on ClinicalTrials.gov (NCT05664282)

## 1 Introduction

Dental crowding is a main indication for orthodontic care, affecting 42%–51% of European children and adolescents, and can negatively impact oral health‐related quality of life and self‐esteem [1, 2, 3]. To address crowding and create space for alignment, orthodontic treatment may involve dental extractions, expansion of the dental arches, proclination of anterior teeth, and interproximal stripping.

Fixed appliances used in orthodontic treatments can be categorised into conventional or self‐ligating, with the latter being either passive or active type. These appliances are attributed specific treatment properties and used with system‐specific or non‐specific arch wires and sequences [4].

Despite extensive research comparing passive self‐ligating bracket systems (PSLB) and conventional bracket systems (CB), large randomised controlled trials (RCTs) are still needed to compare treatment and the subsequent outcomes of PSLB and CB in children and adolescents with crowded teeth [2]. Without sufficient scientific support, practitioners must rely on personal preference and experience for treatment decisions.

Previous RCTs on self‐ligating bracket systems (SLB) and CB assessing occlusal treatment outcomes with the Peer Assessment Rating index [5] (PAR) used the same arch wire sequences and format for both intervention and control during levelling and alignment [6] as well as total treatment [7]. No advantage of either system in reducing the PAR score was found [6, 7].

Dental alignment can be quantitatively evaluated using Little's Iirregularity Index [8] (LII), previously used in both mandibular and maxillary dental arches [9, 10]. A systematic review comparing PSLB and CB on time to initial alignment and overall treatment time found no statistically significant differences [11]. However, due to the clinical heterogeneity of included studies and non‐compelling results, further research is needed to validate these findings [11].

A systematic review found less maxillary incisor proclination associated with PSLB in non‐extraction treatments and less mandibular incisor proclination in extraction treatments compared to CB [12]. Meanwhile, a systematic review on the PSLB, Damon, found no differences in incisor proclination compared to CB but graded the quality of evidence for this outcome as low [13].

A greater increase in mandibular inter‐molar width has been reported for patients treated with PSLB compared to those treated with CB, whereas patients treated with CB demonstrated a greater increase in mandibular inter‐canine width [12]. However, a systematic review on the same studies performed a sensitivity analysis on intermolar width change due to risks of bias, small sample sizes, and clinical heterogeneity of included studies, and found no statistically significant differences [11]. Regarding maxillary lateral expansion, both PSLB and CB have been reported to achieve significant expansion with similar results. However, the use of different expansion protocols and arch wire forms may have influenced the results. More high‐quality RCTs are sought for [11, 13].

Therefore, the overall aim was to assess and compare treatment effects and treatment time of non‐extraction treatments in adolescents with crowding and displaced teeth using either a PSLB (Damon Q) or a CB (Victory APCplus), in a multicentre RCT.

- Primary objective: to assess treatment outcome using weighted PAR (wPAR)
- Secondary objectives: to assess maxillary and mandibular alignment using LII, lateral expansion assessed as intercanine, interpremolar, and intermolar widths, incisor inclination, incisor position, and treatment time.

It was hypothesised that both appliance systems would achieve equal treatment outcomes according to wPAR.

## 2 Materials and Methods

This study is part of the CROWDIT research project assessing outcomes of orthodontic treatments. The Patient Intervention Comparator Outcome (PICO) format framed the research question. Reporting follows the Consolidated Standards of Reporting Trials (CONSORT) checklist [14].

### 2.1 Trial Design and Ethics

This was a multicentre, two‐arm parallel‐group randomised controlled trial (RCT).

The Regional Ethical Review Board, in accordance with the Declaration of Helsinki, and the regional Radiation Committees, approved the trial protocol and informed consent forms (Dnr: 2014/647). The protocol is registered on ClinicalTrials.gov (NCT05664282).

An “intention‐to‐treat” (ITT) approach including all cases as randomised was used to assess effectiveness of being assigned a treatment for the primary wPAR outcomes. Last observation carried forward (LOCF) was used for missing data imputation. The primary wPAR outcomes were complemented with per protocol analyses (PP). Secondary outcomes were assessed with PP for efficacy of receiving treatment. No changes to methods or outcomes were made after trial commencement.

### 2.2 Participants, Settings and Eligibility Criteria

Treatments were performed by four orthodontic specialists (AB, KJ, LP, and MW) at four clinics: two public, one private, and one university clinic in Sweden.

Consecutive eligible patients awaiting approved subsidised orthodontic treatment were recruited at each clinic.

#### 2.2.1 Inclusion Criteria

- 12–17 years of age at treatment initiation.
- Crowded and displaced teeth in one or both arches.
- Sagittal relations within ± one cusp deviation from a normal sagittal relation.
- Overbite ≥ 0 mm.
- Normal transverse relation or minor transverse dental discrepancy.
- Treatment needs 3, 4 or 5 according to the Dental Health Component of the Index of Orthodontic Treatment Need (IOTN DHC) [15].

#### 2.2.2 Exclusion Criteria

Treatment plan including extractions or surgical procedures, need for auxiliary appliances such as a transpalatal bar or Quad Helix; rheumatoid arthritis; missing permanent teeth; impacted teeth; previous orthodontic treatment; ongoing sucking habits; previous trauma to teeth or jaws with subjective, clinical, or radiographic findings; periapical pathology; probing depth of ≥ 5 mm at central incisors or first molars with a calibrated probe, screened at ≥ 4 sites per tooth; visible plaque grade 3 [16]; or communication difficulties.

### 2.3 Randomization, Allocation and Procedures

An allocation sequence was computer‐generated by a statistician using a 1:1 ratio in random permuted blocks of 10 patients, stratified by sex. Each clinic received sealed, opaque, sequentially numbered envelopes marked with either ‘girl’ or ‘boy’.

Patients and their caregivers received verbal and written information about the trial and were invited to participate. After assent and signed consent, an independent staff member opened the consecutive sealed envelope.

#### 2.3.1 Enrolment

After inclusion, clinical examination, medical history, and baseline registrations were completed, the treatment commenced according to treatment protocol.

#### 2.3.2 Baseline Analysis

Space discrepancy was assessed on baseline study casts using a digital calliper (Mitutoyo Manufacturing Co. Ltd., Japan). Segments from mesial contact points of central incisors to distal contact point of lateral incisors, to mesial contact points of first molars were added, and the mesiodistal widths of each tooth were subtracted. Study casts were scanned (at Ortolab, Czestochowa, Poland) into 3D models (O3DM, OrthoLab, DDP‐Ortho software version 2.6.2021–2.14.2023) and used to register IOTN DHC [15], overbite (mm), overjet (mm), sagittal and transversal relations inspired by Björk [17]. Cephalometric analysis was conducted using the computer program Facad (Ilexis AB, Linköping, Sweden). All study casts and 3D models were analysed by LBM, and cephalometric measurements were made by an independent orthodontist with four years of experience (KB).

### 2.4 Treatment Protocol

Treatment goals were bilateral Class I relationship, normal vertical and transverse relations, and aligned teeth. The treatment protocols were developed based on manufacturers' recommendations.

All patients received routine oral hygiene instructions and professional teeth cleaning. Buccal surfaces were etched with 35% phosphoric acid and applied with adhesive in dry conditions using lip and cheek retractor, cotton rolls, dry tip and saliva ejectors.

The PSLB group received Damon Q with 0.022 variable torque (Ormco Corporation, Orange, California, USA) bonded with Greengloo. Damon arch form was used for levelling and alignment, with the following archwire sequence: 0.014 (or 0.013) CuNiTi, 0.018 CuNiTi, and 0.014 × 0.025 (or 0.018 × 0.025) CuNiTi. Control intervals were 8–10 weeks.

The CB group received Victory low profile APC plus adhesive with 0.022 MBT standard torque (3M St Paul, Minnesota, USA). 3M OrthoForm III (Ovoid) arch form was used for levelling and alignment, with the following arch wire sequence: 0.016 (or preceded by 0.012 or 0.014) HANT, 0.019 × 0.025 (or 0.014 × 0.025) HANT. Control intervals were ≥ 6 weeks.

Both groups concluded treatment with the same 0.019 × 0.025 stainless steel (ss) archwire of ovoid form. Archwire advancement occurred when full bracket slot engagement was achieved. Bite disarticulation was placed if required.

Study casts and cephalometric radiographs were created at baseline (T0), at completed alignment (T1, signified by insertion of the first 0.019 × 0.025 ss wire) and at end of active treatment (T2, after debond). Veraviewepocs 3D F40 unit (J. Morita Corp., Kyoto, Japan), Planmeca 3D Mid (Planmeca Oy, Helsinki, Finland), Planmeca Promax 2D (Planmeca Oy, Helsinki, Finland) and Orthoceph OC100D (GE Healthcare, Tuusula, Finland) were used for radiographic imaging.

### 2.5 Blinding

Blinding was not possible for clinicians or patients. The assessment and rating of treatment outcomes on study casts, 3D models and cephalometric radiographs were made using coded materials by LBM and KB who were not involved in the treatment of patients.

## 3 Outcomes

### 3.1 Primary Outcome

Occlusal treatment outcome assessed as wPAR [5] score reduction and percentage reduction post treatment on digital models.

### 3.2 Secondary Outcomes

#### 3.2.1 Assessed on Digital Models

- Alignment from canine to canine in maxillary and mandibular dental arches during overall treatment (T0‐T2) using LII [8].
- Lateral expansion (mm) during alignment (T0‐T1), post alignment (T1‐T2) and T0‐T2, measured as width between the cusp tips of canines, buccal cusp tips of first and second premolars, and mesiobuccal cusp tips of molars. If attrition/abrasion, the measurement point was placed at the centre of that surface.

#### 3.2.2 Assessed on Digital Cephalometric Radiographs

- Central incisor inclination and position from T0–T1, T1–T2 and T0–T2 in the maxillary and mandibular dental arches measured as interincisal angle (°), Ils/NL (°), Ili/ML (°), upper central incisor to NA (°, mm), lower central incisor to NB (°, mm).

Treatment time assessed in months from T0–T1 and T0–T2.

### 3.3 Sample Size Calculation

Based on previous findings, a total of 130 patients (65 per treatment group) were required to demonstrate a mean difference (MD) of 2.5 PAR scores with standard deviations (SD) of 4.27 and 5.56, at a power of 80% and a significance level of 0.05, based on a two‐sided t‐test [6].

### 3.4 Statistical Analysis

The statistical analysis was advised by a statistician.

Descriptive and statistical analyses were performed using SPSS software (IBM SPSS Statistics for Windows, version 28.0.1.1 [14], IL, USA).

Intra‐group changes were analysed with paired samples *t*‐test, and inter‐group comparisons with independent samples Student's *t*‐test (*t*‐test), with 95% confidence interval (CI). Effect of appliance system on all outcomes was controlled for the effect of the variable *orthodontic clinic* in a two‐way analysis of variance (ANOVA). Residuals were tested for normal distribution. Interaction effects between factors were managed by dividing the data file by clinics, controlling for normal distribution of each treatment group, and then conducting a one‐way ANOVA within each clinic. Shapiro–Wilk's test and *Q*–*Q* plots were used for assessing normal distribution.

If Levene's test for equality of variances was significant in *t*‐tests, the adjusted results were used; for the ANOVA, a logarithmised variable (Log10) was applied. If normality was not met, or Levene's test was significant for logarithmised data, a Mann–Whitney *U* exact test was employed per clinic.

Statistical significance was set at *p* < 0.05.

### 3.5 Method Error Analysis

Intra‐ and inter‐rater examiner reliability was assessed with the intraclass correlation coefficient (ICC) with a 95% CI, based on a two‐way mixed‐effects model and absolute agreement [18]. Inter‐rater reliability for cephalometric measurements was conducted by KB and KJ; for space discrepancy by LBM and LP; and for wPAR ratings and 3D model measurements by LBM and KJ. ICC for intra‐ and inter‐rater analyses of performed ratings show moderate to excellent reliability, presented in Table S1.

## 4 Results

**FIGURE 1:**
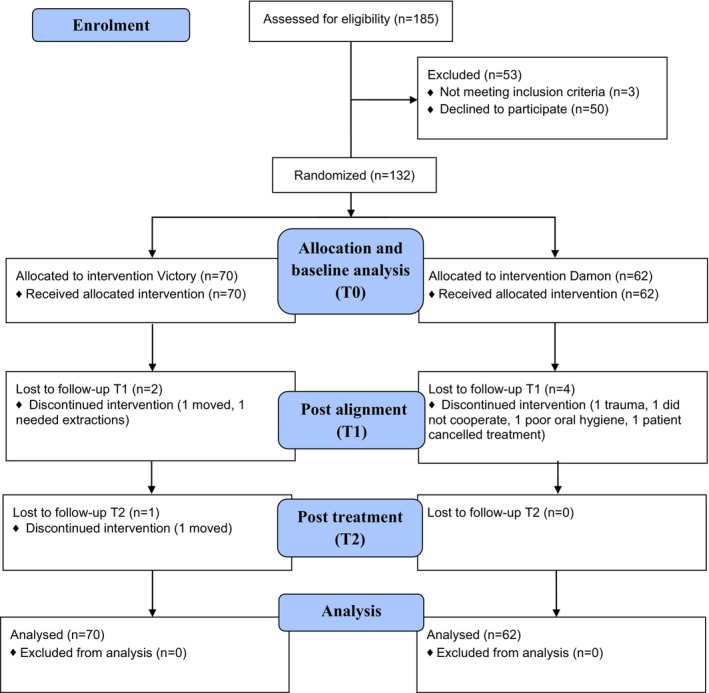
CONSORT flow diagram, based on an intention to treat approach, including all participants irrespective of treatment success or completion.

Recruitment commenced in April 2016 and ended earlier than planned in September 2020 due to the Covid‐19 pandemic. A total of 132 patients received random allocation treatment (PSLB, *n* = 62; CB, *n* = 70); seven treatments were discontinued during the study (see Figure 1, CONSORT flow diagram, based on ITT analysis). Demographics, clinical and cephalometric features at baseline are presented in Table 1.

**TABLE 1:** Demographics, clinical and cephalometric features at baseline.

	CB	PSLB	Total sample
Mean age (SD)	14.61 (1.45)	14.73 (1.48)	14.67 (1.46)
Sex girls/boys, *n*	39/31	34/28	73/59
DHC IOTN grade 3/4/5, *n*	14/56/0	18/43/1	32/99/1
Overjet, mean (mm) (SD)	3.59 (1.60)	3.58 (1.84)	3.58 (1.71)
Overbite, mean (mm) (SD)	3.35 (1.62)	3.90 (1.88)	3.61 (1.76)
Sagittal relation: Class I/Class II/Class III, *n*	48/21/1	39/23/0	87/44/1
Transversal relation, *n*			
Normal/crossbite/scissors bite/crossbite and scissors bite	25/39/6/0	32/25/4/1	57/64/10/1
Space discrepancy upper jaw, mean (mm) (SD)	−3.17 (2.90)	−2.61 (2.34)	−2.91 (2.66)
Space discrepancy lower jaw, mean (mm) (SD)	−2.79 (2.30)	−2.02 (2.32)	−2.43 (2.33)
Maxillary transversal width, mean (mm) (SD)			
13–23 cusp tips	34.07 (2.61)	33.71 (2.90)	33.90 (2.75)
14–24 buccal cusp tips	39.66 (2.68)	39.76 (2.34)	39.71 (2.52)
15–25 buccal cusp tips	44.92 (2.97)	45.05 (2.82)	44.98 (2.89)
16–26 mesiobuccal cusp tips	50.59 (2.99)	50.24 (2.97)	50.43 (2.98)
Mandibular transversal width, mean (mm) (SD)			
33–43 cusp tips	25.69 (2.33)	26.16 (1.88)	25.90 (2.14)
34–44 buccal cusp tips	32.90 (2.46)	33.38 (2.55)	33.12 (2.51)
35–45 buccal cusp tips	38.20 (2.93)	38.73 (2.63)	38.45 (2.79)
36–46 mesiobuccal cusp tips	44.40 (2.91)	44.29 (3.07)	44.35 (2.98)
Cephalometric analysis			
ANB, mean (°) (SD)	2.57 (2.23)	2.74 (2.24)	2.65 (2.23)
Upper central incisor inclination to NA, mean (°) (SD)	21.51 (7.03)	19.27 (9.86)	20.46 (8.51)
Lower central incisor inclination to NB, mean (°) (SD)	23.59 (7.24)	22.01 (6.96)	22.84 (7.13)
Upper central incisor position to NA, mean (mm) (SD)	4.44 (2.64)	3.47 (3.53)	3.98 (3.12)
Lower central incisor position to NB, mean (mm) (SD)	4.02 (2.70)	3.37 (2.47)	3.71 (2.60)
ILs/NL, mean (°) (SD)	109.52 (6.71)	107.70 (9.33)	108.67 (8.07)
ILi/ML, mean (°) (SD)	93.74 (7.47)	92.43 (7.47)	93.13 (7.47)
Interincisal angle, mean (°) (SD)	132.33 (10.99)	135.99 (14.22)	134.05 (12.69)

*Note:* No statistically significant differences between the groups (*p* > 0.05). Total sample (132 patients, 70 CB, 62 PSLB).

Abbreviations: ANB, sagittal inter‐jaw relation; CB, conventional bracket system; DHC IOTN, Dental Health Component of the Index of Orthodontic Treatment Need; ILi/ML, lower incisor inclination relative to mandibular base; ILs/NL, upper incisor inclination relative to maxillary base; Interincisal angle, angle between upper and lower central incisors; ML, mandibular line; mm, millimetre; *n*, number of cases; NA, nasion to subnasal line; NB, nasion to supramental line; NL, nasal line; PSLB, passive self‐ligating bracket system; SD, standard deviation.

Treatment commenced in the maxillary dental arch in 127 patients (PSLB, *n* = 59; CB, *n* = 68) and in the mandibular arch in five patients (PSLB, *n* = 3; CB, *n* = 2). The first dental arch was bonded a mean of 5.6 months (SD 3.87) before the second arch, with a non‐statistically significant (NS) difference between the treatment groups. The first 0.019x0.025 ss arch wire was placed in the upper arch for 84 patients (PSLB, *n* = 37; CB, *n* = 47), in both upper and lower arches at the same appointment for 25 patients (PSLB, *n* = 12; CB, *n* = 13), and in the lower arch for 13 patients (PSLB: 7, CB: 6). All patients were bonded to at least first molars. One patient insisted on only bonding the upper arch (PSLB, *n* = 1), one insisted on debonding the upper arch after alignment (CB, *n* = 1). Ten patients received palatal incisor bite turbos (PSLB, *n* = 7; CB, *n* = 3). Reasons for missing intermediate and endpoint data are displayed in Table S2.

The numbers of maxillary/mandibular central incisors bonded with Damon brackets of standard torque were (38/46), high torque (14/−), and low torque (10/14), respectively. The corresponding torque values were standard (+15°/−3°), high (+22°/−3°), and low (+2°/−11°). Victory brackets used on upper/lower central incisors had a +17°/−6° torque.

### 4.1 Intergroup Analysis

Differences in wPAR score, wPAR score reduction, wPAR percentage reduction, and LII were NS (Tables 2 and S3).

**TABLE 2:** (a) Intergroup comparison of wPAR score at baseline and post treatment, wPAR score reduction and wPAR score percentage reduction during treatment using an independent samples *t*‐test, ITT LOCF analysis. (b) Intergroup comparison of LII at baseline, post treatment, and LII reduction using a PP analysis and independent samples *t*‐test.

(a)										
Treatment	*n*	Mean	SD	Independent samples *t*‐test				Cohens *d*	95% CI of effect size	
				Mean intergroup difference	95% CI of the difference		Significance (two‐sided *p*)			
					Lower	Upper			Lower	Upper
wPAR score, T0										
CB	70	30.31	9.73	1.86	−1.45	5.18	0.268	0.194	−0.149	0.536
PSLB	62	28.45	9.46							
wPAR score, T2										
CB	70	6.24	7.63	−0.10	−2.38	2.19	0.934	−0.014	−0.356	0.327
PSLB	62	6.34	5.28							
wPAR score reduction, T0–T2										
CB	70	−24.07	12.12	−1.96	−5.99	2.07	0.338	−0.168	−0.510	0.175
PSLB	62	−22.11	11.18							
wPAR score percentage reduction, T0–T2										
CB	70	77.78%	0.25	3.35%	−5.03	11.74	0.430	0.138	−0.205	0.480
PSLB	62	74.43%	0.23							

(b)										
Treatment	*n*	Mean	SD	Independent samples *t*‐test				Cohens *d*	95% CI of effect size	
				Mean intergroup difference	95% CI of the difference					
					Lower	Upper	Significance (two‐sided *p*)		Lower	Upper
LII upper arch, T0										
CB	65	10.10	3.76	0.56	−0.67	1.79	0.370	0.163	−0.193	0.519
PSLB	57	9.53	3.02							
LII lower arch, T0										
CB	67	7.03	2.65	0.67	−0.33	1.67	0.192	0.238	−0.116	0.592
PSLB	57	6.37	3.00							
LII upper arch, T2										
CB	66	1.02	0.45	−0.09	−0.27	0.09	0.338	−0.173	−0.526	0.181
PSLB	58	1.11	0.57							
LII lower arch, T2										
CB	66	1.10	0.64	0.00	−0.22	0.22	0.987	−0.003	−0.356	0.350
PSLB	58	1.11	0.59							
LII upper arch reduction, T0–T2										
CB	64	−9.02	3.68	−0.58	−1.81	0.65	0.350	−0.171	−0.528	0.187
PSLB	57	−8.44	3.07							
LII lower arch reduction, T0–T2										
CB	66	−5.95	2.72	−0.66	−1.67	0.35	0.197	−0.235	−0.590	0.121
PSLB	57	−5.29	2.92							

*Note:* No statistically significant intergroup differences (*p* > 0.05).

Abbreviations: CB, conventional bracket system; CI, confidence interval; ITT, intention to treat analysis; LII, Little's irregularity index; LOCF, last observation carried forward; *n*, number of cases; NS, non‐significant; PP, per protocol analysis; PSLB, passive self‐ligating bracket system; SD, standard deviation; T0, baseline; T0–T2; overall treatment; T2, post treatment; wPAR, weighted Peer Assessment Rating.

Lateral expansion T0–T1 was greater in both dental arches for the PSLB group (*p* ≤ 0.019), while intergroup differences T0–T2 were NS (Table 3). The ANOVA analysis supports an effect of appliance system on lateral expansion (Table S4).

**TABLE 3:** Intergroup comparison of lateral expansion (mm) during alignment, post alignment and overall treatment using a PP and independent samples t‐test.

	*n*	ΔT1–T0					Effect size 95% CI			*n*	ΔT2–T1					Effect size 95% CI			*n*	ΔT2–T0					Effect size 95% CI		
		Mean (SD)	Independent samples *t*‐test 95% CI								Mean (SD)	Independent samples *t*‐test 95% CI								Mean (SD)	Independent samples *t*‐test 95% CI						
			Mean difference	*p*	Lower	Upper	Cohen's *d*	Lower	Upper			Mean difference	*p*	Lower	Upper	Cohen's *d*	Lower	Upper			Mean difference	*p*	Lower	Upper	Cohen's *d*	Lower	Upper
13–23 cusp tips																											
CB	62	0.01 (1.80)	−1.35	**< 0.001**	−2.05	−0.66	−0.72	−1.09	−0.34	63	0.33 (0.72)	0.72	**< 0.001**	0.40	1.03	0.84	0.46	1.22	64	0.41 (1.90)	−0.55	0.130	−1.26	0.16	−0.28	−0.64	0.08
PSLB	55	1.36 (1.98)								56	−0.39 (0.98)								57	0.96 (2.06)							
14–24 buccal cusp tips																											
CB	64	2.77 (1.64)	−1.21	**< 0.001**	−1.76	−0.66	−0.80	−1.17	−0.42	63	0.01 (0.75)	0.84	**< 0.001**	0.51	1.17	0.94	0.56	1.32	66	2.85 (1.90)	−0.32	0.304	−0.91	0.28	−0.19	−0.54	0.17
PSLB	56	3.98 (1.37)								56	−0.83 (1.03)								58	3.16 (1.46)							
15–25 buccal cusp tips																											
CB	63	2.89 (1.83)	−0.48	0.138	−1.13	0.16	−0.28	−0.65	0.09	62	−0.02 (0.95)	0.68	**< 0.001** ^a^	0.31	1.06	0.67	0.29	1.04	66	2.88 (2.03)	0.06	0.864	−0.62	0.74	0.03	−0.33	0.39
PSLB	53	3.37 (1.62)								55	−0.70 (1.10)								56	1.73 (0.23)							
16–26 mesiobuccal cusp tips																											
CB	64	0.63 (1.49)	−0.48	0.073	−1.01	0.05	−0.33	−0.70	0.03	63	0.25 (1.28)	0.61	**0.009** ^a^	0.15	1.07	0.49	0.12	0.86	66	0.81 (1.56)	0.00	1.000	−0.55	0.55	0.00	−0.35	0.35
PSLB	55	1.11 (1.41)								55	−0.36 (1.21)								58	0.81 (1.51)							
33–43 cusp tips																											
CB	64	0.91 (1.81)	−0.27	0.397	−0.91	0.36	−0.16	−0.52	0.21	63	0.13 (0.81)	0.21	0.099	−0.04	0.47	0.31	−0.06	0.67	66	1.10 (1.98)	0.05	0.890	−0.62	0.71	0.03	−0.33	0.38
PSLB	55	1.18 (1.66)								56	−0.08 (0.55)								57	1.05 (1.70)							
34–44 buccal cusp tips																											
CB	64	1.81 (1.67)	−0.75	**0.019**	−1.37	−0.12	−0.44	−0.80	−0.07	63	−0.07 (1.10)	0.42	**0.029** ^a^	0.04	0.79	−0.41	0.04	0.77	66	1.75 (1.86)	−0.44	0.227	−1.15	0.27	−0.44	−0.80	−0.07
PSLB	55	2.56 (1.77)								55	−0.48 (0.93)								58	2.19 (2.14)							
35–45 buccal cusp tips																											
CB	64	2.05 (1.74)	−0.76	**0.018**	−1.39	−0.13	−0.44	−0.80	−0.07	63	−0.25 (1.29)	0.54	**0.024**	0.07	1.01	0.42	0.06	0.78	66	1.86 (2.37)	−0.16	0.688	−0.92	0.61	−0.07	−0.42	0.28
PSLB	56	2.81 (1.74)								56	−0.80 (1.30)								58	2.02 (1.89)							
36–46 mesiobuccal cusp tips																											
CB	64	−0.19 (1.24)	−0.47	0.063	−0.95	0.02	−0.35	−0.72	0.02	63	0.26 (1.41)	0.50	0.069	−0.04	1.05	0.34	−0.03	0.71	66	0.06 (1.65)	0.21	0.589	−0.55	0.96	0.10	−0.26	0.45
PSLB	53	0.27 (1.43)								53	−0.24 (1.55)								58	−0.14 (2.54)							

*Note: p*‐values in bold are statistically significant (*p* < 0.05).

Abbreviations: Δ, change in transversal width; CB, conventional bracket system; CI, confidence interval; mm, millimetre; *n*, number of cases; *p*, *p*‐value; PP, per protocol analysis; PSLB, passive self‐ligating bracket system; SD, standard deviation; T0, baseline; T1, post alignment; T2, post treatment.

^a^ Effect of appliance system was not detected in the ANOVA (Table S4).

Central incisor proclination was greater T0–T1, and interincisal angle decreased more in the CB group (*p* ≤ 0.016) (Table 4). Intergroup differences for change in inclination T0–T2 were NS (Table 4). Intergroup differences in central incisor position T0–T1 and T0–T2 were NS (Table 4). The effect of appliance system on maxillary changes in inclination was not detected in the ANOVA, while an effect on changes in mandibular central incisor inclination and interincisal angle was supported (Table S5).

**TABLE 4:** Intergroup comparison of anterior expansion during alignment, post alignment and overall treatment using a PP and independent samples *t*‐test.

	*n*	ΔT1–T0					Effect size 95% CI			*n*	ΔT2–T1					Effect size 95% CI			*n*	ΔT2–T0					Effect size 95% CI		
		Mean (SD)	Independent samples *t*‐test 95% CI								Mean (SD)	Independent samples *t*‐test 95% CI								Mean (SD)	Independent samples *t*‐test 95% CI						
			Mean difference	*p*	Lower	Upper	Cohen's *d*	Lower	Upper			Mean difference	*p*	Lower	Upper	Cohen's *d*	Lower	Upper			Mean difference	*p*	Lower	Upper	Cohen's *d*	Lower	Upper
Upper central incisor to NA (*°*)																											
CB	63	7.80 (6.80)	2.89	**0.040** ^a^	0.14	5.63	0.38	0.02	0.75	63	−1.14 (4.15)	−1.32	0.064	−2.72	0.08	−0.35	−0.71	0.02	66	6.85 (6.46)	1.73	0.211	−1.00	4.47	0.23	−0.12	0.59
PSLB	55	4.91 (8.25)								55	0.18 (3.42)								57	5.11 (8.50)							
Lower central incisor to NB (*°*)																											
CB	63	6.35 (4.94)	2.28	**0.015**	0.45	4.10	0.46	0.09	0.82	63	1.10 (4.82)	−3.26	**< 0.001**	−5.05	−1.48	−0.67	−1.04	−0.29	66	7.44 (5.70)	−0.84	0.433	−2.95	1.28	−0.14	−0.50	0.21
PSLB	55	4.07 (5.07)								55	4.36 (4.97)								57	8.28 (6.03)							
Upper central incisor to NA (mm)																											
CB	63	1.86 (2.18)	0.52	0.229	−0.33	1.36	−0.14	−0.50	0.21	63	−0.21 (1.33)	−0.12	0.572	−0.52	0.29	−0.10	−0.46	0.26	66	1.65 (2.01)	0.42	0.298	−0.37	1.20	0.19	−0.17	0.55
PSLB	55	1.35 (2.47)								55	−0.09 (0.87)								56	1.24 (2.39)							
Lower central incisor to NB (mm)																											
CB	63	1.89 (1.68)	0.35	0.241	−0.24	0.93	0.22	−0.15	0.58	63	0.28 (1.06)	−0.58	**0.003**	−0.96	−0.20	−0.57	−0.94	−0.19	66	2.10 (1.66)	−0.29	0.325	−0.86	0.29	−0.18	−0.54	0.18
PSLB	55	1.55 (1.48)								54	0.86 (0.99)								56	2.39 (1.51)							
ILsNL (*°*)																											
CB	63	8.02 (6.55)	3.09	**0.022** ^a^	0.45	5.74	0.43	0.06	0.79	63	−1.50 (4.02)	−1.44	**0.039**	−2.81	−0.07	−0.39	−0.75	−0.02	66	6.76 (6.55)	1.88	0.158	−0.74	4.50	0.26	−0.10	0.61
PSLB	55	4.93 (7.95)								55	−0.06 (3.40)								57	4.88 (8.11)							
ILiML (*°*)																											
CB	63	6.30 (4.92)	2.23	**0.016**	0.42	4.03	0.45	0.08	0.82	63	1.41 (4.71)	−2.77	**0.003**	−4.56	−0.98	−0.57	−0.93	−0.20	66	7.59 (5.59)	−0.55	0.597	−2.60	1.49	−0.10	−0.45	0.26
PSLB	55	4.08 (4.98)								55	4.18 (5.10)								57	8.14 (5.82)							
Interincisal angle (*°*)																											
CB	63	−14.15 (9.04)	−4.97	**0.007**	−8.57	−1.36	−0.50	−0.87	−0.14	63	0.22 (6.32)	4.54	**< 0.001**	2.42	6.66	0.78	0.41	1.16	66	−14.05 (8.91)	−0.67	0.750	−4.38	3.04	−0.07	−0.42	0.29
PSLB	55	−9.18 (10.72)								55	−4.32 (5.13)								57	−13.37 (11.82)							

*Note: p*‐values in bold are statistically significant (*p* < 0.05).

Abbreviations: Δ, change in anterior expansion; CB, conventional bracket system; CI, confidence interval; ILi/ML (°), lower incisor inclination relative to mandibular base; ILs/NL (°), upper incisor inclination relative to maxillary base; Interincisal angle, angle between upper and lower central incisors; ML, mandibular line; mm, millimetre; *n*, number of cases; NA, nasion to subnasal line; NB, nasion to supramental line; NL, nasal line; NS, non‐significant; *p*, *p*‐value; PP, per protocol analysis; PSLB, passive self‐ligating bracket system; SD, standard deviation; T0, baseline; T1, post alignment, T2, post treatment.

^a^ Effect of appliance system was not detected in the ANOVA (Table S5).

Time to alignment and total treatment time were NS between groups. The analysis was conducted after consulting a statistician and reaching a consensus to remove one distinct outlier in the PSLB group (57.63 months total treatment time due to travel and living abroad) (Table 5). No effect of appliance system on treatment time was detected in the ANOVA (Table S6).

**TABLE 5:** Intergroup comparison of treatment time as time to alignment, post alignment time and total treatment time in months, using independent samples *t*‐test on PP.

Group	*n*	Mean	SD	Independent samples *t*‐test				Cohen's *d*	95% CI Cohen's *d*
				Mean difference	95% CI		*p*		
					Lower	Upper			
T0 to T1									
CB	65	12.12	4.98	−1.51	−3.42	0.40	0.121	−0.29	−0.65/0.08
PSLB	55	13.63	5.59						
T1 to T2									
CB	65	9.45	5.90	−1.02	−3.10	1.07	0.337	−0.18	−0.54/0.18
PSLB	55	10.47	5.56						
T0 to T2									
CB	67	21.74	7.57	−2.61	−5.23	0.02	0.051	−0.36	−0.71/−0.002
PSLB	57	24.35	7.10						

*Note:* No statistically significant intergroup differences (*p* > 0.05). Excluding a PSLB outlier (57.63 months).

Abbreviations: CB, conventional bracket system; CI, confidence interval; *n*, number of cases; NS, non‐significant; *p*, *p*‐value; PP, per protocol analysis; PSLB, passive self‐ligating bracket system; SD, standard deviation; T0 to T1, time to alignment; T1 to T2, post alignment; T0 to T2, total treatment time.

Complementary PP analysis of intergroup differences for wPAR outcomes was NS (Tables S7 and S8). All measurements for transversal width and cephalometric analysis at time points T0, T1 and T2 are presented in Table S9. Intergroup comparison of treatment time including the outlier is presented in Table S10 for transparency.

### 4.2 Intragroup Analysis

All intragroup changes T0–T1 and T0–T2 were statistically significant (*p* < 0.001), except for lateral expansion between maxillary canines in the CB group (NS T0–T1, T0–T2) and lateral expansion between mandibular first molars in both treatment groups (NS T0–T1, T0–T2).

#### 4.2.1 Harms

No harms were noted other than soft tissue sores from brackets, plaque accumulation and associated gingivitis.

## 5 Discussion

In this RCT, both PSLB and CB groups demonstrated great improvement after orthodontic non‐extraction treatments in adolescents with crowded teeth and severe tooth displacements assessed with wPAR. In accordance with our hypothesis, no significant wPAR inter‐group difference was found. Dental crowding and tooth displacement were alleviated through posterior and anterior expansion in both groups. Overall, similar and successful occlusal outcomes according to wPAR and LII were achieved using both appliance systems. Further, total treatment times were similar with NS intergroup differences.

### 5.1 Lateral and Anterior Expansion

The observed expansion patterns formed during the alignment phase differed between the groups in accordance with arch wire format. During T0–T1, the broad Damon arch wire format was used for the PSLB group, and a greater lateral expansion was observed. In contrast, the CB group was treated with the ovoid arch wire format, and a greater mandibular incisor proclination was observed. The suggested effect of arch wire format is further supported by the subsequent reduction of intergroup differences after identical 0.019x0.025 ss ovoid arch wires were placed in both groups at T1. Moreover, a recent RCT assessed the impact of arch wire form on transverse and incisal dental arch changes during levelling and alignment, comparing broad Damon arch wires to ovoid wires in patients with crowding treated without extractions. Both groups were bonded with Victory brackets. The Damon arch wire group showed a significant increase in certain dental arch widths and less mandibular incisor proclination and horizontal advancement compared to the Ovoid group [19]. However, in the current study, the mandibular inter molar expansion did not show significant intra‐ or inter‐group differences during alignment. This is notable since the broad Damon arch form widens posteriorly. However, the lateral expansion at molars might not have been fully achieved during the alignment phase. Furthermore, the current study did not assess the type of tooth movement achieved during lateral expansion. Previous studies have associated PSLB with more buccal inclination of the posterior teeth compared to CB [20, 21]. Hence, the reduction in lateral expansion observed from T1 to T2 in the PSLB group could be attributed to both a root torque correction and an archwire contraction induced by switching from the previously used broad Damon arch wire during alignment to an ovoid 0.019 × 0.025 stainless steel wire at T1.

Previous RCTs on crowding comparing PSLB and CB paired with identical arch wires found no intergroup differences in dental arch dimensional change in non‐extraction treatments [22, 23] or in extraction treatments [24]. In accordance with this rationale, previous clinical trials and RCTs found that SLB combined with broad Damon arch wires increased transverse maxillary inter‐premolar width [25] and mandibular intermolar width [9, 26] to a greater extent than CB combined with relatively narrower wires. However, a recent study comparing PSLB, active self‐ligating brackets (ASLB), and CB paired with identical arch wires until ligation of 0.019 × 0.025 ss still found a greater lateral expansion increase in both PSLB and ASLB groups compared to the CB group, as measured on CBCT [21]. Likewise, a larger inter‐molar width was observed during alignment in an SLB group compared to CB although the same arch wire form was used [27]. This indicates an influence of other factors than the shape of arch wires on final expansion outcomes.

The clinical relevance of the dimensional changes observed in the current study could be discussed in relation to gained dental arch perimeter, treatment stability and the risk of adverse effects. A mathematical model on gained arch perimeter through expansion treatments indicates that most arch space is gained through incisor advancement compared to lateral expansion, and more through lateral expansion anteriorly at canines than at the molars [28]. An increased inter‐canine width has been associated with increased risk of relapse, making a more pronounced posterior lateral premolar expansion during alignment a more desirable feature for orthodontic non‐extraction treatments in patients with dental crowding [29]. Nevertheless, a retrospective study on the stability of dental arch dimensions 6 years post‐treatment reported relapse in the premolar region after non‐extraction treatments of moderate dental crowding with the Damon system and canine‐to‐canine retainers [30]. Furthermore, there is a potential risk of adverse effects on alveolar bone such as fenestrations and dehiscences, and the subsequent potential risk of gingival recessions from excessive arch expansion [31], stressing the importance of individual treatment planning based on scientific evidence.

### 5.2 Treatment Time and Alignment

The difference in time to alignment between treatment groups was NS. Total treatment time was shorter for the CB group but NS after removing the PSLB outlier. The outlier's extended treatment time (57.63 months) was considered unrelated to the orthodontic treatment and removed after consultation with a statistician and a reached consensus within the research group. A meta‐analysis of four clinical trials found no significant intergroup differences in time to alignment; yet, the total treatment duration was significantly longer with CB, by an average of 2.01 months [32]. Since the sample size in the current study was not calculated based on this variable, the results should be interpreted with caution.

The amount of irregularity has been discussed as a challenging circumstance for PSLB [33]. In cases with severe irregularity, the bracket might not be able to engage the wire during the first stages of levelling if the bracket slot cannot be closed. The patients in the current study had pronounced irregularity (Table 2b), potentially pinching the wire, reducing some claimed advantages of PSLB [4]. Previous studies have found the alignment rate to be influenced by the initial amount of irregularity [10, 24, 26]. A previous study on non‐extraction treatments found that PSLB corrects irregularities 2.7 times faster than CB in patients with initial moderate LII < 5, as opposed to LII > 5 [26]. Another study on PSLB and CB in extraction patients with a mean initial LII of 12 found NS inter‐group differences in time to alignment [24]. However, contradicting results [23, 34] highlight the influence of other factors and so the complexity of orthodontic treatment.

According to the Damon treatment protocol, bonding of both jaws at the same occasion is recommended. In this study, bonding of both jaws at the same time or on different occasions was optional. It is possible the results had been different for the PSLB if this recommendation had been pursued.

## 6 Strengths and Limitations

### 6.1 Sample Size and Randomization

The global Covid‐19 pandemic prematurely cancelled enrolment, limiting the sample size and causing uneven treatment group sizes. Despite this, successful randomisation of known variables was achieved (Table 1), possibly because of block randomisation. RCTs reduce bias through randomisation of unknown confounders. Due to vast distances, randomisation to orthodontic clinic was not possible. Therefore, the outcomes were controlled for the effect of orthodontic clinic in ANOVA analyses.

### 6.2 Generalisability

The multicentre design, involving four orthodontic clinics in three geographic regions, enhances the generalisability of the results of orthodontic treatment without extractions using assessed bracket systems (and protocols recommended by manufacturers) in populations of similar age, diagnosis, and clinical characteristics. However, Damon Q has been further developed by manufacturers, why the use of newer generations may yield different results.

### 6.3 Bracket Systems

The use of different sets of arch wires per treatment group has been recognised as a confounder in previous studies on SLB and CB [22, 35]. This study was designed to compare bracket systems used according to the manufacturers' recommendations (current at the time of study initiation), to enhance the clinical relevance of study results.

Bite turbos were used during initial treatment in ten patients, potentially affecting maxillary incisal inclination to some extent. However, eight patients were from the same clinic and therefore likely accounted for in the ANOVA, which controlled for the effect of orthodontic clinic. Nevertheless, the maxillary incisor inclination result is to be interpreted with this considered.

### 6.4 Missing Data

The discontinued treatments were few (Figure 1), and the intermediate missing data (Table S2) was judged unrelated to treatment allocation. A detailed description of data analysis is essential for interpreting results [14, 36]. An ITT approach was used for the primary wPAR outcomes to represent the effectiveness of being assigned a treatment and to avoid overestimation of results [37]. Patients were included and analysed as randomised despite treatment failure or discontinuation. LOCF was used for data imputation [38]. Complementary PP analysis, including only participants who completed the protocol as randomised with available outcome assessments, was conducted as a sensitivity analysis to the ITT results. Remaining treatment outcomes were analysed with PP for the efficacy of having received treatment, reasoning this may be of interest in everyday clinical practice.

Overall, both ITT and PP can provide biased estimates. ITT uses assumptions for imputing data, and PP risks selection bias and distortion of randomised known and unknown confounders [37, 39]. However, both methods are considered relevant and valid, with different scopes and interpretations [37]. Therefore, due to missing data, the presented results should be interpreted with awareness of the strengths and weaknesses of ITT and PP analysis.

### 6.5 Future Studies

Future research may investigate the effect of broad and narrow arch wires from start to end of treatment, analyse potential adverse effects and the stability of expansion treatments.

## 7 Conclusions

The aim of the present study was to assess and compare treatment effects and treatment time of PSLB and CB in non‐extraction treatments in adolescents with crowding and displaced teeth. The study supports the hypothesis of similar and successful treatment outcomes according to wPAR and LII. Moreover, different expansion patterns were observed during alignment, with more lateral expansion using PSLB and broad wires, compared to more anterior expansion using a CB combined with ovoid wires. All treatments were concluded with identical stainless‐steel wires, and the final lateral and anterior expansion outcomes were similar in both groups. These findings could further be used to individualise treatment protocols based on desired treatment effects during treatment.

## Author Contributions

Each author has made substantial contributions to the conception and design, acquisition of data, or analysis and interpretation of data. They have either been involved in drafting the manuscript or revising it critically for important intellectual content. All authors have given final approval of the version to be published and have participated sufficiently in the work to take public responsibility for appropriate portions of the content. Each author agrees to be accountable for all aspects of the work, ensuring that questions related to the accuracy or integrity of any part of the work are appropriately investigated and resolved.

## Ethics Statement

The Regional Ethical Review Board in accordance with the Declaration of Helsinki, and the regional Radiation Committees have approved the trial protocol and informed consent forms. Informed consent was obtained prior to inclusion in the study.

## Conflicts of Interest

The authors declare no conflicts of interest.

## Supporting information


Data S1.

Table S1.

Table S2.

Table S3.

Table S4.

Table S5.

Table S6.

Table S7.

Table S8.

Table S9.

Table S10.

## Data Availability

The data that support the findings of this study are available from the corresponding author upon reasonable request.
