# Supplementary material for: Treatment Effects and Treatment Time in Adolescents With Crowded and Displaced Teeth Treated With Fixed Appliance Systems Without Extractions: A Multi‐Centre Randomised Controlled Trial
Source: Orthod Craniofac Res. 2025 Jul 23;28(6):929–42. doi: 10.1111/ocr.70005 (PMC12603669; doi:10.1111/ocr.70005)
Supplement: Supplementary file 2 — Table S1. [file OCR-28-929-s009.docx]

| Supplementary Table 1 (S1): Intra- and Inter-rater reliability: Intraclass Correlation Coefficients, Single Rating, Absolute-Agreement, 2-Way Mixed-Effects Model | | | | | | | | |
| --- | --- | --- | --- | --- | --- | --- | --- | --- |
| **Variable** |  |  | **ICC** | | |  |  | |
|  |  | **95% CI** | | |  | | **95% CI** | |
|  | **Inter rater** | **Lower** | | **Upper** | **Intra rater** | | **Lower** | **Upper** |
| wPAR T0 | 0.897 | 0.782 | | 0.953 | 0.975 | | 0.945 | 0.989 |
| wPAR score difference | 0.906 | 0.801 | | 0.957 | 0.967 | | 0.926 | 0.985 |
| LII ÖK | 0.978 | 0.914 | | 0.995 | 0.981 | | 0.960 | 0.991 |
| LII UK | 0.964 | 0.819 | | 0.991 | 0.992 | | 0.983 | 0.996 |
| Maxillary space discrepancy | 0.837 | 0.486 | | 0.957 | 0.942 | | 0.883 | 0.972 |
| Mandibular space discrepancy | 0.803 | 0.359 | | 0.948 | 0.951 | | 0.900 | 0.976 |
| Width 13-23 | 0.999 | 0.997 | | 1.00 | 0.997 | | 0.993 | 0.998 |
| Width 16-26 | 0.908 | 0.669 | | 0.976 | 0.996 | | 0.992 | 0.998 |
| Width 33-43 | 0.999 | 0.998 | | 1.00 | 0.993 | | 0.985 | 0.997 |
| Width 36-46 | 0.934 | 0.758 | | 0.983 | 0.988 | | 0.962 | 0.995 |
| ILs/NL | 0.990 | 0.960 | | 0.997 | 0.975 | | 0.947 | 0.988 |
| ILi/ML | 0.936 | 0.768 | | 0.984 | 0.966 | | 0.930 | 0.984 |
| Upper central incisor to NA° | 0.993 | 0.972 | | 0.998 | 0.972 | | 0.942 | 0.986 |
| Lower central incisor to NB° | 0.893 | 0.648 | | 0.972 | 0.970 | | 0.938 | 0.986 |
| Upper central incisor to NA (mm) | 0.988 | 0.953 | | 0.997 | 0.975 | | 0.949 | 0.988 |
| Lower central incisor to NB (mm) | 0.893 | 0.648 | | 0.972 | 0.993 | | 0.986 | 0.997 |
| Interincisal angle | 0.952 | 0.830 | | 0.988 | 0.978 | | 0.954 | 0.989 |
| Note: ICC results indicate a range of poor to excellent reliability.  Inter-rater reliability for cephalometric measurements was conducted by (…) and author (…), space discrepancy between authors (…) and (…), and all other measurements by authors (…) and (…). ICC for wPAR were assessed on 25 cases. The inter-rater reliability analysis for LII, transversal width, space discrepancy and cephalometric measurements were assessed on 10 cases, all intra-rater reliability for these measurements was assessed on 30 cases.  Abbreviations: ICC, Intraclass Correlation Coefficient; CI, Confidence Interval; wPAR, weighted Peer Assessment Rating; LII, Little’s Irregularity Index; NA: Nasion to subnasal line; NB, nasion to supramental line; ILs/NL, Upper incisor inclination relative to maxillary base; ILi/ML, Lower incisor inclination relative to mandibular base; NL, Nasal Line; ML, Mandibular Line; Interincisal angle, the angle between upper and lower central incisors. | | | | | | | | |
