# Supplementary material for: Treatment Effects and Treatment Time in Adolescents With Crowded and Displaced Teeth Treated With Fixed Appliance Systems Without Extractions: A Multi‐Centre Randomised Controlled Trial
Source: Orthod Craniofac Res. 2025 Jul 23;28(6):929–42. doi: 10.1111/ocr.70005 (PMC12603669; doi:10.1111/ocr.70005)
Supplement: Supplementary file 3 — Table S2. [file OCR-28-929-s007.docx]

| Supplementary Table 2 (S2): Reasons for missing intermediate and end point data | | | |
| --- | --- | --- | --- |
| **Study models T1**  *5 CB, 6 PSLB* | **Study models T2**  *4 CB, 2 PSLB* | **Cephalograms T1**  *7 CB, 7 PSLB* | **Cephalograms T2**  *4 CB, 4 PSLB* |
| 1 trauma | 1 lost | 1 trauma | 1 trauma |
| 1 moved | 2 moved | 2 moved | 2 moved |
| 1 needed extractions | 1 needed extractions | 1 needed extractions | 1 needed extractions |
| 1 did not cooperate poor oral hygiene | 1 did not cooperate - oral hygiene | 1 did not cooperate – poor oral hygiene | 1 did not cooperate - oral hygiene |
| 5 missed | 1 cancelled | 6 missed/lost | 2 missed/lost |
| 2 cancelled |  | 2 cancelled | 1 cancelled |
|  |  | 1 not performed - to close to debond |  |
| Abbreviations: T1, post alignment; T2, post treatment; CB, conventional bracket system; PSLB, passive self-ligating bracket system. | | | |
