# Supplementary material for: Treatment Effects and Treatment Time in Adolescents With Crowded and Displaced Teeth Treated With Fixed Appliance Systems Without Extractions: A Multi‐Centre Randomised Controlled Trial
Source: Orthod Craniofac Res. 2025 Jul 23;28(6):929–42. doi: 10.1111/ocr.70005 (PMC12603669; doi:10.1111/ocr.70005)
Supplement: Supplementary file 4 — Table S3. [file OCR-28-929-s003.docx]

| Supplementary Table 3 (S3) a: Effect of appliance system on wPAR posttreatment, wPAR score reduction, wPAR score percentage reduction, while controlling for the effect of *orthodontic* *clinic* with a two-way ANOVA analysis, based on the ITT LOCF analysis | | | | | | | | |
| --- | --- | --- | --- | --- | --- | --- | --- | --- |
|  | Treatment group | n | Estimated marginal mean | Std error | 95% CI of the mean estimate | | p | η_p_^2^ |
|  |  |  |  |  | Lower | Upper |  |  |
| wPAR score posttreatment † | CB | 70 | 6.66 | 0.89 | 4.90 | 8.43 | 0.720 | 0.001 |
|  | Damon | 62 | 6.22 | 0.84 | 4.56 | 7.89 |  |  |
| wPAR score reduction | CB | 70 | -23.75 | 1.62 | -26.95 | -20.56 | 0.198 | 0.013 |
|  | Damon | 62 | -20.88 | 1.52 | -23.89 | -17.87 |  |  |
| wPAR score percent reduction ‡ | CB | 70 | 75.46 % | 3.15 | 69.23 | 81.68 | 0.760 | 0.001 |
|  | Damon | 62 | 74.13 % | 2.96 | 68.27 | 80.00 |  |  |
| Supplementary Table 3 (S3) b: Effect of appliance system on LII, when controlling for the effect of *orthodontic* *clinic* with a two-way ANOVA analysis, based on the PP analysis | | | | | | | | |
| LII upper arch posttreatment | CB | 66 | 1.02 | 0.08 | 0.85 | 1.18 | 0.404 | 0.006 |
|  | PSLB | 58 | 1.11 | 0.08 | 0.96 | 1.27 |  |  |
| LII lower arch posttreatment | CB | 66 | 1.11 | 0.10 | 0.92 | 1.30 | 0.607 | 0.002 |
|  | PSLB | 58 | 1.18 | 0.09 | 1.00 | 1.36 |  |  |
| LII upper arch reduction | CB | 64 | -8.80 | 0.56 | -9.91 | -7.69 | 0.406 | 0.006 |
|  | PSLB | 57 | -8.16 | 0.53 | -9.20 | -7.11 |  |  |
| LII lower arch reduction | CB | 66 | -5.86 | 0.43 | -6.72 | -5.0 | 0.081 | 0.026 |
|  | PSLB | 57 | -4.80 | 0.41 | -5.62 | -3.98 |  |  |
| Note: p-values in bold are statistically significant (p<0.05).  † Significant Levene’s test for variable, result controlled with log10. All clinics: NS).  ‡ Significant Levene’s test for the log10 variable, results controlled with Mann Whitney U test per clinic. Clinic A: **p=0.029**, all other NS.  Abbreviations: wPAR, weighted Peer Assessment Rating; LII, Little’s irregularity index; ANOVA, analysis of variance; ITT, intention to treat analysis; LOCF, last observation carried forward; PP, per protocol analysis; n, number of cases; Std error, standard error; CI, confidence interval; p, p-value; PP, per protocol; η_p_^2^, partial eta squared; CB, conventional bracket system; PSLB, passive self-ligating bracket system; NS, non-significant. | | | | | | | | |
