# Supplementary material for: Treatment Effects and Treatment Time in Adolescents With Crowded and Displaced Teeth Treated With Fixed Appliance Systems Without Extractions: A Multi‐Centre Randomised Controlled Trial
Source: Orthod Craniofac Res. 2025 Jul 23;28(6):929–42. doi: 10.1111/ocr.70005 (PMC12603669; doi:10.1111/ocr.70005)
Supplement: Supplementary file 5 — Table S4. [file OCR-28-929-s011.docx]

| Supplementary Table 4: Effect of appliance system on lateral expansion (mm) when controlling for the effect of orthodontic clinic during alignment, post alignment and overall treatment, using a two-way ANOVA on PP analysis. | | | | | | | | | | | | | | | | | | | | | | | | | | |  |
| --- | --- | --- | --- | --- | --- | --- | --- | --- | --- | --- | --- | --- | --- | --- | --- | --- | --- | --- | --- | --- | --- | --- | --- | --- | --- | --- | --- |
|  |  | ΔT1-T0 | |  | 95% CI of EM | | |  | |  | |  | ΔT2-T1 |  | 95% CI of EM | |  |  |  | ΔT2-T0 |  | | 95% CI of EM | |  |  | |
|  | n | EM | | Standard  Error | Lower | Upper | | p | | η_p_^2^ | | n | EM | Standard  Error | Lower | Upper | p | η_p_^2^ | n | EM | Standard  Error | | Lower | Upper | p | η_p_^2^ | |
| **13-23 cusp tips** | | | | | | | | | | | | | | | | | | | | | | | | | | |  |
| CB | 62 | 0.11 | | 0.31 | -0.50 | 0.73 | | **0.005** | | 0.071 | | 63 | †a | | | | | | 64 | 0.51 | 0.32 | | -0.13 | 1.14 | 0.355 | 0.008 | |
| PSLB | 55 | 1.34 | | 0.29 | 0.76 | 1.92 | |  |  |  |  | 56 |  |  |  |  |  |  | 57 | 0.91 | 0.30 | | 0.32 | 1.51 |  |  |  |
| **14-24 buccal cusp tips** | | | | | | | | | | | | | | | | | | | | | | | | | | |  |
| CB | 64 | †b | | | | | | | | | | 63 | ‡a | | | | | | 66 | †c | | | | | | |  |
| PSLB | 56 |  |  |  |  |  |  |  |  |  |  | 56 |  |  |  |  |  |  | 58 |  |  |  |  |  |  |  |  |
| **15-25 buccal cusp tips** | | | | | | | | | | | | | | | | | | | | | | | | | | |  |
| CB | 63 | 2.81 | | 0.28 | 2.26 | 3.37 | | 0.150 | | 0.019 | | 62 | ‡b | | | | | | 66 | 3.19 | 0.30 | | 2.60 | 3.80 | 0.474 | 0.004 | |
| PSLB | 53 | 3.38 | | 0.27 | 2.84 | 3.92 | |  |  |  |  | 55 |  |  |  |  |  |  | 56 | 2.89 | 0.29 | | 2.32 | 3.46 |  |  |  |
| **16-26 mesiobuccal cusp tips** | | | | | | | | | | | | | | | | | | | | | | | | | | |  |
| CB | 64 | 0.60 | | 0.23 | 0.13 | 1.06 | | 0.095 | | 0.025 | | 63 | ‡c | | | | | | 66 | 1.20 | | 0.24 | 0.72 | 1.68 | 0.399 | 0.006 | |
| PSLB | 55 | 1.14 | | 0.22 | 0.70 | 1.59 | |  |  |  |  | 55 |  |  |  |  |  |  | 58 | 0.92 | | 0.23 | 0.47 | 1.37 |  |  |  |
| **33-43 cusp tips** | | | | | | | | | | | | | | | | | | | | | | | | | | |  |
| CB | 64 | 0.85 | | 0.27 | 0.31 | 1.39 | | 0.878 | | 0.000 | | 63 | 0.19 | 0.11 | -0.03 | 0.41 | 0.194 | 0.015 | 66 | 1.10 | 0.29 | | 0.52 | 1.70 | 0.560 | 0.003 | |
| PSLB | 55 | 0.91 | | 0.26 | 0.39 | 1.42 | |  |  |  |  | 56 | -0.01 | 0.11 | -0.22 | 0.20 |  |  | 57 | 0.86 | 0.28 | | 0.31 | 1.42 |  |  |  |
| **34-44 buccal cusp tips** | | | | | | | | | | | | | | | | | | | | | | | | | | |  |
| CB | 64 | ‡d | | | | | | | | | | 63 | 0.00 | 0.16 | -0.32 | 0.32 | 0.056 | 0.033 | 66 | 2.23 | 0.32 | | 1.60 | 2.85 | 0.618 | 0.002 | |
| PSLB | 55 |  |  |  |  |  |  |  |  |  |  | 55 | -0.43 | 0.15 | -0.74 | -0.13 |  |  | 58 | 2.01 | 0.30 | | 1.42 | 2.60 |  |  |  |
| **35-45 buccal cusp tips** | | | | | | | | | | | | | | | | | | | | | | | | | | |  |
| CB | 64 | 2.07 | 0.29 | | 1.50 | | 2.63 | | **0.045** | | 0.035 | 63 | 0.03 | 0.20 | -0.36 | 0.42 | **0.014** | 0.053 | 66 | 2.13 | 0.34 | | 1.46 | 2.81 | 0.865 | 0.000 | |
| PSLB | 56 | 2.86 | 0.27 | | 2.23 | | 3.40 | |  |  |  | 56 | -0.64 | 0.19 | -1.01 | -0.28 |  |  | 58 | 2.21 | 0.32 | | 1.57 | 2.85 |  |  |  |
| **36-46 mesiobuccal cusp tips** | | | | | | | | | | | | | | | | | | | | | | | | | | |  |
| CB | 64 | §a | | | | | | | | | | 63 | §b | | | | | | 66 | 0.29 | 0.33 | | -0.36 | 0.94 | 0.682 | 0.001 | |
| PSLB | 53 |  |  |  |  |  |  |  |  |  |  | 53 |  |  |  |  |  |  | 58 | 0.11 | 0.31 | | -0.51 | 0.72 |  |  |  |
| Note: p-values in bold are statistically significant (p<0.05).  † Significant Levene’s test. Mann Whitney U test per clinic: (†a) clinic A **p=0.014**, clinic B **p=0.010**, other clinics: NS. (†b) clinic B: **p=0.009**, clinic C: **p<0.001**, clinics A and D (NS) (†c) clinic B: **p=0.041**, other clinics: NS.  ‡ Interaction effect between factors. Mann Whitney U test per clinic: (‡a) clinic A: **p=0.010**, C: **p=0.006**, D: **p=0.038,** Clinic B: NS. (‡b) all clinics: NS. (‡c) all clinics NS. (‡d) Clinic B: **p=0.010**, clinic C: **p=0.006**, clinic D: **p=0.010**, Clinic A=NS.  § Non normal distribution. Mann Whitney U test per clinic: (§a) all clinics NS. (§b) all clinics NS.  Abbreviations: ANOVA, analysis of variance; PP, per protocol analysis; Δ, change in transversal width; T0, baseline; T1 post alignment, T2, post treatment; CI, confidence interval; n, number of cases; EM, estimated marginal mean; p, p-value; η_p_^2^, effect size as partial eta squared; CB, conventional bracket system; PSLB, passive self-ligating bracket system; NS, non-significant. | | | | | | | | | | | | | | | | | | | | | | | | | | |  |
