# Supplementary material for: Treatment Effects and Treatment Time in Adolescents With Crowded and Displaced Teeth Treated With Fixed Appliance Systems Without Extractions: A Multi‐Centre Randomised Controlled Trial
Source: Orthod Craniofac Res. 2025 Jul 23;28(6):929–42. doi: 10.1111/ocr.70005 (PMC12603669; doi:10.1111/ocr.70005)
Supplement: Supplementary file 6 — Table S5. [file OCR-28-929-s002.docx]

| Supplementary Table 5 (S5): Effect of appliance system on anterior expansion assessed with cephalometric analysis, when controlling for the effect of orthodontic clinic, during alignment, post alignment and overall treatment, using a two-way ANOVA on PP | | | | | | | | | | | | | | | | | | | | | | | |
| --- | --- | --- | --- | --- | --- | --- | --- | --- | --- | --- | --- | --- | --- | --- | --- | --- | --- | --- | --- | --- | --- | --- | --- |
|  |  | ΔT1-T0 |  | 95% CI of EM | | |  |  |  | ΔT2-T1 |  | 95% CI of  EM | |  |  |  | ΔT2-T0 |  | 95% CI of EM | |  | |  |
|  | n | EM | Standard  Error | Lower | Upper | | p | η_p_^2^ | n | EM | Standard  Error | Lower | Upper | p | η_p_^2^ | n | EM | Standard  Error | Lower | Upper | p | | η_p_^2^ |
| **Upper central incisor inclination to NA (°)** | | | | | | | | | | | | | | | |  |  |  |  |  |  | |  |
| CB | 63 | 7.02 | 1.21 | 4.62 | 9.42 | | 0.298 | 0.010 | 63 | -1.09 | 0.63 | -2.33 | 0.15 | 0.152 | 0.19 | 66 | 6.08 | 1.19 | 3.73 | 8.43 | 0.658 | | 0.002 |
| PSLB | 55 | 5.27 | 1.15 | 2.99 | 7.56 | |  |  | 55 | 0.15 | 0.60 | -1.03 | 1.33 |  |  | 57 | 5.35 | 1.13 | 3.11 | 7.59 |  |  |  |
| **Lower central incisor inclination to NB (°)** | | | | | | | | | | | | | | | |  |  |  |  |  |  | |  |
| CB | 63 | 6.04 | 0.76 | 4.53 | 7.55 | | **0.005** | 0.069 | 63 | †a | | | | | | 66 | 6.50 | 0.92 | 4.67 | 8.32 | 0.244 | | 0.012 |
| PSLB | 55 | 3.04 | 0.72 | 1.60 | 4.47 | |  |  | 55 |  |  |  |  |  |  | 57 | 7.98 | 0.88 | 6.25 | 9.72 |  |  |  |
| **Upper central incisor to NA (mm)** | | | | | | | | | | | | | | | |  |  |  |  |  |  | |  |
| CB | 63 | 1.78 | 0.38 | 1.03 | 2.53 | | 0.568 | 0.003 | 63 | -0.34 | 0.18 | -0.70 | 0.02 | 0.261 | 0.011 | 66 | 1.44 | 0.35 | 0.74 | 2.15 | 0.972 | | 0.000 |
| PSLB | 55 | 1.48 | 0.36 | 0.77 | 2.20 | |  |  | 55 | -0.05 | 0.17 | -0.40 | 0.29 |  |  | 56 | 1.43 | 0.35 | 0.73 | 2.13 |  |  |  |
| **Lower central incisor to NB (mm)** | | | | | | | | | | | | | | | | | | | | | |  | |
| CB | 63 | 1.60 | 0.24 | 1.12 | 2.08 | | 0.316 | 0.009 | 63 | †b | | | | | | 66 | 1.83 | 0.25 | 1.34 | 2.32 | 0.143 | | 0.019 |
| PSLB | 55 | 1.26 | 0.23 | 0.81 | 1.72 | |  |  | 54 |  |  |  |  |  |  | 56 | 2.34 | 0.25 | 1.85 | 2.83 |  |  |  |
| **ILs/NL (°)** | | | | | | | | | | | | | | | |  |  |  |  |  |  | |  |
| CB | 63 | 7.22 | 1.18 | 4.89 | | 9.56 | 0.203 | 0.015 | 63 | -1.57 | 0.62 | -2.79 | -0.35 | 0.076 | 0.028 | 66 | 5.85 | 1.17 | 3.54 | 8.16 | 0.602 | | 0.002 |
| PSLB | 55 | 5.14 | 1.12 | 2.91 | | 7.36 |  |  | 55 | -0.04 | 0.59 | -1.21 | 1.12 |  |  | 57 | 5.01 | 1.11 | 2.80 | 7.21 |  |  |  |
| **ILi/ML (°)** | | | | | | | | | | | | | | | |  |  |  |  |  |  | |  |
| CB | 63 | 6.06 | 0.78 | 4.52 | | 7.60 | **0.009** | 0.060 | 63 | ‡a | | | | | | 66 | 6.69 | 0.91 | 4.89 | 8.49 | 0.322 | | 0.009 |
| PSLB | 55 | 3.21 | 0.74 | 1.75 | | 4.68 |  |  | 53 |  |  |  |  |  |  | 57 | 7.94 | 0.87 | 6.22 | 9.66 |  |  |  |
| **Interincisal angle (°)** | | | | | | | | | | | | | | | |  |  |  |  |  |  | |  |
| CB | 63 | -13.00 | 1.61 | -16.20 | | -9.81 | **0.039** | 0.038 | 63 | †c | | | | | | 66 | -12.20 | 1.67 | -15.52 | -8.88 | 0.661 | | 0.002 |
| PSLB | 55 | -8.36 | 1.53 | -11.40 | | -5.32 |  |  | 55 |  |  |  |  |  |  | 57 | -13.22 | 1.60 | -16.38 | -10.05 |  |  |  |
| Note: p-values in bold are statistically significant (p<0.05).  † Interaction effect between factors. Mann Whitney U test per clinic: (†a) clinic B: **p=0.019**, D: **p=0.010**, other clinics: NS. (†b) clinic B: **p=0.005**; clinic D: **p= 0.016**, other clinics: NS. (†c) clinic B: **p<0.001**, clinic D: **p=0.003**, other clinics: NS.  ‡ Interaction effect between factors. One way ANOVA per clinic: (‡a) clinic B: **p=0.008**, clinic D: **p=0.004**, other clinics: NS.  Abbreviations: ANOVA, analysis of variance; PP, per protocol analysis; Δ, change in transversal width; T0, baseline; T1 post alignment, T2, post treatment; mm, millimetre; CI, confidence interval; n, number of cases; EM, estimated marginal mean; p, p-value; η_p_^2^, effect size as partial eta squared; NA: nasion to subnasal line; NB, nasion to supramental line; ILs/NL, upper incisor inclination relative to maxillary base; ILi/ML, lower incisor inclination relative to mandibular base; NL, nasal line; ML, mandibular line; Interincisal angle, angle between upper and lower central incisors; CB, conventional bracket system; PSLB, passive self-ligating bracket system; NS, non-significant. | | | | | | | | | | | | | | | | | | | | | | | |

dc
