# Supplementary material for: Treatment Effects and Treatment Time in Adolescents With Crowded and Displaced Teeth Treated With Fixed Appliance Systems Without Extractions: A Multi‐Centre Randomised Controlled Trial
Source: Orthod Craniofac Res. 2025 Jul 23;28(6):929–42. doi: 10.1111/ocr.70005 (PMC12603669; doi:10.1111/ocr.70005)
Supplement: Supplementary file 7 — Table S6. [file OCR-28-929-s001.docx]

| Supplementary Table 6 (S6): Effect of appliance system on treatment time (months), when controlling for the effect of orthodontic clinic, during alignment, post alignment and overall treatment, using a two-way ANOVA on PP | | | | | | | | |
| --- | --- | --- | --- | --- | --- | --- | --- | --- |
|  |  | |  |  | **95% CI of EM** | |  |  |
|  | n | | EM | Standard  Error | Lower | Upper | p | η_p_^2^ |
| **T0-T1** | | | | | | | | |
| CB | 65 | †a | | | | | | |
| PSLB | 55 |  |  |  |  |  |  |  |
| **T1-T2** | | | | | | | | |
| CB | 65 | †b | | | | | | |
| PSLB | 55 |  |  |  |  |  |  |  |
| **T0-T2** | | | | | | | | |
| CB | 67 | †c | | | | | | |
| PSLB | 57 |  |  |  |  |  |  |  |
| Note: p-values in bold are statistically significant (p<0.05). Excluding a PSLB outlier (57.63 months).  † Interaction effect between factors. Mann Whitney U test per clinic: (†a) clinic A: **p=0.011**, other clinics: NS. (†b) all clinics: NS. (†c) Clinic A: **p=0.010**, other clinics: NS.  Abbreviations: ANOVA, analysis of variance; PP, per protocol analysis; n, number of cases; EM, estimated marginal mean; CI, confidence interval; p, p-value significance; CB, conventional bracket system; PSLB, passive self-ligating bracket system; η_p_^2^, effect size as partial eta squared; T0-T1, time to alignment; T1-T2; post alignment; T0-T2, total treatment time; NS, non-significant. | | | | | | | | |
