# Supplementary material for: Treatment Effects and Treatment Time in Adolescents With Crowded and Displaced Teeth Treated With Fixed Appliance Systems Without Extractions: A Multi‐Centre Randomised Controlled Trial
Source: Orthod Craniofac Res. 2025 Jul 23;28(6):929–42. doi: 10.1111/ocr.70005 (PMC12603669; doi:10.1111/ocr.70005)
Supplement: Supplementary file 8 — Table S7. [file OCR-28-929-s008.docx]

| Supplementary Table 7 (S7): Intergroup comparison of wPAR score at baseline and post treatment, wPAR score reduction and wPAR score percentage reduction during treatment using an independent samples t-test (PP) | | | | | | | | | | | |
| --- | --- | --- | --- | --- | --- | --- | --- | --- | --- | --- | --- |
|  | Treatment outcome | n | Mean | SD | Independent samples t-test | | | |  |  | |
|  |  |  |  |  |  | 95% CI of the difference | |  |  | 95% CI of effect size | |
|  |  |  |  |  | Mean difference | Lower | Upper | p | Cohens d | Lower | Upper |
| wPAR score pretreatment, T0 | CB | 67 | 30.31 | 9.79 | 1.33 | -2.06 | 4.72 | 0.439 | 0.139 | -0.213 | 0.491 |
|  | PSLB | 58 | 28.98 | 9.26 |  |  |  |  |  |  |  |
| wPAR score posttreatment, T2 | CB | 66 | 4.73 | 4.21 | -1.36 | -3.01 | 0.29 | 0.106 | -0.293 | -0.647 | 0.062 |
|  | PSLB | 58 | 6.09 | 5.08 |  |  |  |  |  |  |  |
| wPAR score reduction T0-T2 | CB | 66 | -25.53 | 10.87 | 2.63 | -1.21 | 6.47 | 0.177 | 0.224 | -0.110 | 0.598 |
|  | PSLB | 58 | -22.90 | 10.67 |  |  |  |  |  |  |  |
| wPAR score percent reduction, T0-T2 | CB | 66 | 82.49% | 16.87 | 5.60% | -0.81% | 12.01% | 0.086 | 0.311 | -0.044 | 0.665 |
|  | PSLB | 58 | 76.89% | 19.19 |  |  |  |  |  |  |  |
| Note: p-values in bold are statistically significant (p<0.05).  Abbreviations: PP, per protocol; wPAR, weighted Peer Assessment rating; LII, Little’s irregularity index; T0, baseline; T2, post treatment; T0-T2; overall treatment; n, number of cases; SD, standard deviation; CI, confidence interval; wPAR, weighted Peer Assessment rating; p, p-value; CB, conventional bracket system; PSLB, passive self-ligating bracket system; NS, non-significant. | | | | | | | | | | | |
