# Supplementary material for: Treatment Effects and Treatment Time in Adolescents With Crowded and Displaced Teeth Treated With Fixed Appliance Systems Without Extractions: A Multi‐Centre Randomised Controlled Trial
Source: Orthod Craniofac Res. 2025 Jul 23;28(6):929–42. doi: 10.1111/ocr.70005 (PMC12603669; doi:10.1111/ocr.70005)
Supplement: Supplementary file 9 — Table S8. [file OCR-28-929-s010.docx]

| Supplementary Table 8 (S8): Effect of appliance system on wPAR posttreatment, wPAR score reduction, wPAR score percentage reduction, when controlling for the effect of *orthodontic clinic* using a two-way ANOVA analysis, based on the PP analysis | | | | | | | | |
| --- | --- | --- | --- | --- | --- | --- | --- | --- |
|  | Treatment group | n | Estimated marginal mean | Std error | 95% CI of the mean estimate | | p | η_p_^2^ |
|  |  |  |  |  | Lower | Upper |  |  |
| wPAR score posttreatment † | CB | 66 | 5.45 | 0.66 | 4.15 | 6.76 | 0.475 | 0.004 |
|  | PSLB | 58 | 6.10 | 0.63 | 4.86 | 7.34 |  |  |
| wPAR score reduction | CB | 66 | -24.69 | 1.60 | -21.53 | -27.85 | 0.145 | 0.018 |
|  | PSLB | 58 | -21.46 | 1.51 | -18.47 | -24.56 |  |  |
| wPAR score percent reduction ‡ | CB | 66 | 78.99 % | 2.49 | 74.06 | 83.93 | 0.378 | 0.007 |
|  | PSLB | 58 | 75.95 % | 2.36 | 71.27 | 80.64 |  |  |
| Note: No statistically significant differences between the groups (p>0.05).  † Significant Levene’s test for variable, result controlled with log10 (NS).  ‡ Significant Levene’s test for log10 variable, Mann Whitney U test per clinic: all results were NS within each clinic  Abbreviations: wPAR, weighted Peer Assessment Rating; ANOVA, analysis of variance; PP, per protocol analysis; n, number of cases; Std error, standard error; CI, confidence interval; p, p-value; PP, per protocol; η_p_^2^, partial eta squared; CB, conventional bracket system; PSLB, passive self-ligating bracket system; NS, non-significant. | | | | | | | | |
