# Supplementary material for: Treatment Effects and Treatment Time in Adolescents With Crowded and Displaced Teeth Treated With Fixed Appliance Systems Without Extractions: A Multi‐Centre Randomised Controlled Trial
Source: Orthod Craniofac Res. 2025 Jul 23;28(6):929–42. doi: 10.1111/ocr.70005 (PMC12603669; doi:10.1111/ocr.70005)
Supplement: Supplementary file 10 — Table S9. [file OCR-28-929-s004.docx]

| Supplementary Table 9 (S9): Transverse measurements and cephalometric analysis at T0, T1 and T2 (PP) | | | | | | | | |
| --- | --- | --- | --- | --- | --- | --- | --- | --- |
| **TRANSVERSE MEASUREMENTS** | | | | | | | | |
|  | Appliance |  | **T0** |  | **T1** |  | **T2** |  |
|  | system | n | Mean (SD) | n | Mean (SD) | n | Mean (SD) |  |
| Maxillary transversal measurements | | | | | | | |  |
| 13-23 cusp tips | CB | 65 | 34.29 (2.43) | 64 | 34.40 (1.74) | 66 | 34.80 (1.53) |  |
|  | PSLB | 57 | 33.85 (2.63) | 56 | 35.30 (1.66) | 58 | 34.83 (1.53) |  |
| 14-24 buccal cusp tips | CB | 67 | 39.82 (2.61) | 64 | 42.58 (1.87) | 66 | 42.73 (1.68) |  |
|  | PSLB | 58 | 39.60 (2.20) | 56 | 43.67 (1.75) | 58 | 42.76 (1.60) |  |
| 15-25 buccal cusp tips | CB | 67 | 45.07 (2.95) | 63 | 47.84 (2.08) | 66 | 48.00 (1.78) |  |
|  | PSLB | 58 | 45.87 (4,74) | 55 | 48.73 (2.12) | 58 | 47.95 (1.96) |  |
| 16-26 mesiobuccal cusp tips | CB | 67 | 50.78 (2.92) | 64 | 51.29 (2.50) | 66 | 51.69 (2.08) |  |
|  | PSLB | 58 | 50.23 (3,05) | 55 | 51.53 (2.47) | 58 | 51.05 (2.27) |  |
| Mandibular transversal measurements | | | | | | | | |
| 33-43 cusp tips | CB | 67 | 25.81 (2.23) | 64 | 26.74 (1.42) | 66 | 26.95 (1.29) |  |
|  | PSLB | 57 | 26.05 (1.89) | 56 | 27.15 (1.55) | 58 | 27.02 (1.36) |  |
| 34-44 buccal cusp tips | CB | 67 | 33.15 (2.10) | 64 | 34.88 (1.38) | 66 | 34.94 (1.45) |  |
|  | PSLB | 58 | 33.30 (2.57) | 55 | 36.06 (1.62) | 58 | 35.49 (1.57) |  |
| 35-45 buccal cusp tips | CB | 67 | 38.42 (2.77) | 64 | 40.39 (1.71) | 66 | 40.32 (1.82) |  |
|  | PSLB | 58 | 38.79 (2.70) | 56 | 41.67 (2.08) | 58 | 40.81 (1.94) |  |
| 36-46 mesiobuccal cusp tips | CB | 67 | 44.61 (2.80) | 64 | 44.32 (2.48) | 66 | 44.69 (2.07) |  |
|  | PSLB | 58 | 44.31 (3.16) | 53 | 44.78 (2.93) | 58 | 44.17 (3.05) |  |
| **CEPHALOMETRIC ANALYSIS** | | | | | | | | |
| Upper central incisor to NA (°) | CB | 67 | 21.68 (7.10) | 63 | 29.42 (5.20) | 66 | 28.39 (5.48) |  |
|  | PSLB | 58 | 18.66 (9.57) | 55 | 23.82 (5.86) | 57 | 23.92 (5.48) |  |
| Lower central incisor to NB (°) | CB | 67 | 23.44 (7.19) | 63 | 29.85 (6.79) | 66 | 31.03 (6.04) |  |
|  | PSLB | 58 | 21.80 (7.05) | 55 | 25.90 (6.00) | 57 | 30.31 (5.68) |  |
| Upper central incisor position to NA (mm) | CB | 67 | 4.49 (2.67) * | 63 | 6.38 (2.22) | 66 | 6.12 (2.17) |  |
|  | PSLB | 58 | 3.20 (3.36) * | 55 | 4.64 (2.01) | 56 | 4.48 (1.94) |  |
| Lower central incisor position to NB (mm) | CB | 67 | 3.99 (2.73) | 63 | 5.94 (2.63) | 66 | 6.15 (2.54) |  |
|  | PSLB | 58 | 3.29 (2.47) | 55 | 4.85 (2.17) | 56 | 5.78 (2.16) |  |
| ILs/NL(°) | CB | 67 | 109.52 (6.80) | 63 | 117.42 (5.59) | 66 | 116.13 (6.34) |  |
|  | PSLB | 58 | 107.08 (9.07) | 55 | 111.92 (5.66) | 57 | 112.02 (5.52) |  |
| ILi/ML (°) | CB | 67 | 93.76 (7.47) | 63 | 99.88 (7.35) | 66 | 101.31 (7.01) |  |
|  | PSLB | 58 | 92.37 (7.54) | 55 | 96.54 (7.17) | 57 | 100.70 (6.79) |  |
| Interincisal angle (°) | CB | 67 | 132.40 (11.04) | 63 | 118.28 (9.40) | 66 | 118.33 (8.87) |  |
|  | PSLB | 58 | 136.65 (14.14) | 55 | 127.26 (7.92) | 57 | 122.88 (6.94) |  |
| Note: *Intergroup difference in upper central incisor position to NA in mm (PP) was significant at T0 (mean difference: 1.29, 95% CI 0.022-2.35, **p=0.018**).  Abbreviations: T0, baseline; T1, post alignment, T2, post treatment; CB, conventional bracket system; PSLB, passive self-ligating bracket system; mm, millimetre; PP, per protocol analysis; SD, Standard deviation; n, number of cases; NA, nasion to subnasal line; NB, nasion to supramental line; ILs/NL, upper incisor inclination relative to maxillary base; ILi/ML, lower incisor to inclination relative to mandibular base; NL, nasal line; ML, mandibular line; Interincisal angle, angle between upper and lower central incisors. | | | | | | | | |
