# Supplementary material for: Treatment Effects and Treatment Time in Adolescents With Crowded and Displaced Teeth Treated With Fixed Appliance Systems Without Extractions: A Multi‐Centre Randomised Controlled Trial
Source: Orthod Craniofac Res. 2025 Jul 23;28(6):929–42. doi: 10.1111/ocr.70005 (PMC12603669; doi:10.1111/ocr.70005)
Supplement: Supplementary file 11 — Table S10. [file OCR-28-929-s005.docx]

| Supplementary Table 10 (S10): Intergroup comparison of treatment time as time to alignment, post alignment time and total treatment time in months, using independent samples t-test on PP | | | | | | | | | | | |
| --- | --- | --- | --- | --- | --- | --- | --- | --- | --- | --- | --- |
|  |  |  |  |  | **Independent samples t-test** | | | |  | |  |
|  | **Group** | **n** |  |  |  | 95% CI | |  |  | |  |
|  |  |  | **Mean** | **SD** | **Mean difference** | **Lower** | **Upper** | **p** | **Cohens d** | | **95% CI Cohens d** |
| T0 to T1 | CB | 65 | 12.12 | 4.98 | -1.92 | -3.95 | 0.12 | 0.065 | | -0.340 | -0.70/0.02 |
|  | PSLB | 56 | 14.04 | 6.33 |  |  |  |  |  |  |  |
| T1 to T2 | CB | 65 | 9.45 | 5.90 | -1.21 | -3.30 | 0.89 | 0.256 | | -0.208 | -0.57/0.15 |
|  | PSLB | 56 | 10.66 | 5.69 |  |  |  |  |  |  |  |
| T0 to T2 | CB | 67 | 21.74 | 7.57 | -3.18 | -5.99 | -0.37 | **0.027** | | -0.402 | -0.76/-0.05 |
|  | PSLB | 58 | 24.92 | 8.29 |  |  |  |  |  |  |  |

| Note: p-values in bold are statistically significant (p<0.05). Including outlier (PSLB: 56.7 months)  Abbreviations: PP, per protocol analysis; CI*,* confidence interval; n*,* number of cases; SD, standard deviation; CI, confidence interval; p, p-value; T0 to T1, time to alignment; T1 to T2, post alignment; T0 to T2, total treatment time; CB, conventional bracket system; PSLB, passive self-ligating bracket system; NS, non-significant. |
| --- |
